# Fabrication of Well-Aligned ZnO Nanorods Using a Composite Seed Layer of ZnO Nanoparticles and Chitosan Polymer

**DOI:** 10.3390/ma6104361

**Published:** 2013-09-30

**Authors:** Kimleang Khun, Zafar Hussain Ibupoto, Mohamad S. AlSalhi, Muhammad Atif, Anees A. Ansari, Magnus Willander

**Affiliations:** 1 Physical Electronics and Nanotechnology Division, Department of Science and Technology, Campus Norrkoping, Linkoping University, Norrkoping SE-60174, Sweden; E-Mails: zafar.hussain.ibupoto@liu.se (Z.H.I.); magnus.willander@liu.se (M.W.); 2 Research Chair for Laser Diagnosis of Cancer, King Saud University, Riyadh 11451, Saudi Arabia; E-Mail: malsalhi@ksu.edu.sa; 3 Physics and Astronomy Department, College of Science, King Saud University, Riyadh 11451, Saudi Arabia; E-Mails: atifhull@gmail.com; 4 King Abdullah Institute for Nanotechnology, King Saud University, Riyadh, 11451, Saudi Arabia; E-Mail: aneesaansari@gmail.com

**Keywords:** ZnO nanoparticles, chitosan, ZnO nanorods, well-aligned, low-temperature growth

## Abstract

In this study, by taking the advantage of both inorganic ZnO nanoparticles and the organic material chitosan as a composite seed layer, we have fabricated well-aligned ZnO nanorods on a gold-coated glass substrate using the hydrothermal growth method. The ZnO nanoparticles were characterized by the Raman spectroscopic techniques, which showed the nanocrystalline phase of the ZnO nanoparticles. Different composites of ZnO nanoparticles and chitosan were prepared and used as a seed layer for the fabrication of well-aligned ZnO nanorods. Field emission scanning electron microscopy, energy dispersive X-ray, high-resolution transmission electron microscopy, X-ray diffraction, and infrared reflection absorption spectroscopic techniques were utilized for the structural characterization of the ZnO nanoparticles/chitosan seed layer-coated ZnO nanorods on a gold-coated glass substrate. This study has shown that the ZnO nanorods are well-aligned, uniform, and dense, exhibit the wurtzite hexagonal structure, and are perpendicularly oriented to the substrate. Moreover, the ZnO nanorods are only composed of Zn and O atoms. An optical study was also carried out for the ZnO nanoparticles/chitosan seed layer-coated ZnO nanorods, and the obtained results have shown that the fabricated ZnO nanorods exhibit good crystal quality. This study has provided a cheap fabrication method for the controlled morphology and good alignment of ZnO nanorods, which is of high demand for enhancing the working performance of optoelectronic devices.

## 1. Introduction

Among several compound semiconductors, ZnO is widely used in the development of optoelectronic devices due to its versatile properties, such as a wide direct bad gap of 3.37 eV at room temperature, a high exciton binding energy of 60 meV, an optical grain of approximately 300 cm^−1^ and high mechanical and thermal stability. Recently, one-dimensional ZnO nanostructure has received much focus. The controlled morphology, growth parameters and physical properties of these structures are being intensely discussed by the researchers. Extensive efforts have been made to control the morphology and methods to achieve better alignment and well-controlled morphology of ZnO nanostructures [1]. ZnO has been widely used in several applications such as in catalysis [2], Gratzel-type solar cells [3], short-wavelength light-emitting devices [4,5], transparent conductors [6], chemical sensors [7], and piezoelectric nanomaterials [8]. The use of well-aligned ZnO nanorods in the development of a UV laser [9] has strongly motivated researchers to study the alignment of ZnO nanostructures, such as nanowires/nanorods, because the controlled morphology has a significant effect on the working performance of the nanoscale-based optoelectronics devices. Several growth methods have been utilized for the fabrication of well-aligned 1D ZnO nanostructures such as the vapor-liquid-solid (VLS) technique [9], chemical vapor deposition (CVD) [10,11], electrochemical deposition (ED) [12], and hydrothermal methods [13,14,15,16,17]. The CVD and ED techniques are highly sensitive, with very demanding conditions, including the need for a single crystalline substrate [9,10,11,12]. In addition to this, a catalyst and a high temperature of 890 °C for VLS [9] and 500 °C for CVD [10] are required for the growth of nanorods. However, the hydrothermal approach has several advantages such as, it is cheap and simple, and gives high yield of ZnO on substrate. Highly oriented ZnO micro rods and micro tubes have been fabricated using the hydrothermal method with a hetero-nucleation approach, which provides a higher saturation ratio than a homogeneous solution does [18,19].

Although the hydrothermal method has many advantages compared to the ED, VLS, and CVD methods, yet the properties exhibited by the hydrothermally synthesized ZnO nanorods are not much better than the properties exhibited by the nanorods fabricated by the ED, VLS, and CVD approaches. An X-ray diffraction (XRD) analysis has indicated the precise orientation of the nanorods perpendicular to the substrate by exhibiting only the characteristic diffraction peaks of 002 and 004 for the patterned arrays of the ZnO nanorods using the VLS [9], CVD [10,11], and ED [12] techniques. However, the ZnO nanorods synthesized by the hydrothermal growth method possessed additional diffraction peaks, including (100), (101), and (102) [13,14], which indicates a small deviation in the perpendicular orientation relative to the substrate of some portion of the ZnO nanorods. The control over the orientation, morphology, growth density and aspect ratio of the hydrothermally grown ZnO nanorods/nanowires is still the most debating issue among researchers.

Chitosan has the ability to bind strongly with a negatively charged surface due to its positive charge and can also make gels and complexes with polyanions. It is soluble in different acids and exhibits antibacterial and antifungal responses in addition to exhibiting biosafe and nontoxic properties [20,21]. Semiconductor nanoparticles are attractive to many researchers due to their wonderful optical, electrical, and mechanical properties. Among the several metal oxide nanoparticles, ZnO nanoparticles currently receive a lot of attention due to their significant contributions to applied sciences, such as in the areas of solar energy conversion, varistors, luminescence, photocatalysis, electrostatic dissipative coating, transparent UV protection films, and chemical sensors [22]. Several methods have been used for the preparation of ZnO nanoparticles, including sol-gel [23,24], precipitation [25], hydrothermal [26], and spray pyrolysis [27] methods.

In this paper, the ZnO nanoparticles were synthesized in an ethanolic medium and then mixed with a chitosan solution prepared in 1% acetic acid. The resulting composite was used as a seed layer for the fabrication of vertically aligned ZnO nanorods. We studied the effect of different composites of ZnO nanoparticles and chitosan as a seed on the alignment of the ZnO nanorods by keeping a constant amount of chitosan in the composite. The present study describes the potential applicability of the hybrid composite material as a seed layer for the fabrication of well-aligned ZnO nanorods oriented exactly perpendicular to the growing substrate.

## 2. Results and Discussion

### 2.1. The XRD Study of the ZnO Nanorods Fabricated Using Different Composite Seed Layers of ZnO Nanoparticles in a Chitosan Solution

**Figure 1:**
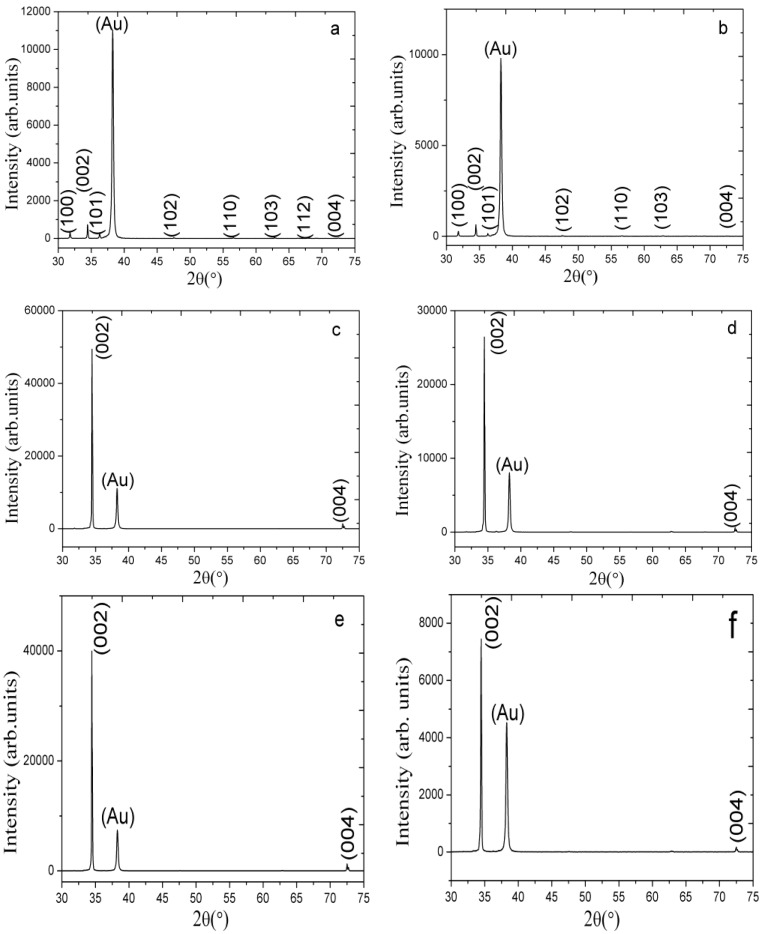
The XRD pattern of the ZnO nanorods grown using the seed solution of ZnO nanoparticles and chitosan with different amounts of ZnO nanoparticles (**a**) 0; (**b**) 10; (**c**) 30; (**d**) 50; (**e**) 70; and (**f**) 90 mg.

The results obtained from the XRD study for the different composite seed layers of ZnO nanoparticles/chitosan for the growth of ZnO nanorods are shown in Figure 1a–f. In each XRD graph, a gold peak is appeared due to the gold layer on the glass substrate. Figure 1a shows the XRD pattern of the grown ZnO nanorods without the use of ZnO nanoparticles in the seed solution and it can be observed that (002) peak is very weak and the orientation of ZnO nanorods is remained a subject of matter. However, Figure 1b shows the (002), (100), (101), (102), (110), and (103) peaks for the ZnO nanorods fabricated using the 10 mg of ZnO nanoparticles/chitosan seed layer. Although the (002) peak is very intense and demonstrates a growth pattern along the *c*-axis, other peaks are also apparent. However, as the quantity of ZnO nanoparticles in the seed solution increases, a dominant growth pattern along only (002) and (004) planes is observed, as shown in Figure 1e. Additionally, the intense growth along the *c*-axis direction suppressed the growth pattern in the other planes, as shown in Figure 1b,d,f. The composite seed layer of ZnO nanoparticles and chitosan strongly enhanced the (002) peak, and the primary growth pattern is observed only along the *c*-axis; this phenomenon is rarely observed when using the hydrothermal growth method.

The XRD study demonstrated a good crystal quality, a wurtzite hexagonal structure and a good orientation of the ZnO nanorods along the *c*-axis. The possible role of chitosan in the seed layer might be as solid gel type support for the firm adhesion of seed particles on the substrate with better nucleation. We have observed during the preparation of seed solution that chitosan provides uniform distribution of ZnO nanoparticles, which further helps in the growth orientation of ZnO nanorods.

### 2.2. The Morphological Study of the ZnO Nanoparticles/Chitosan Composite Seed Layer-Coated ZnO Nanorods

**Figure 2:**
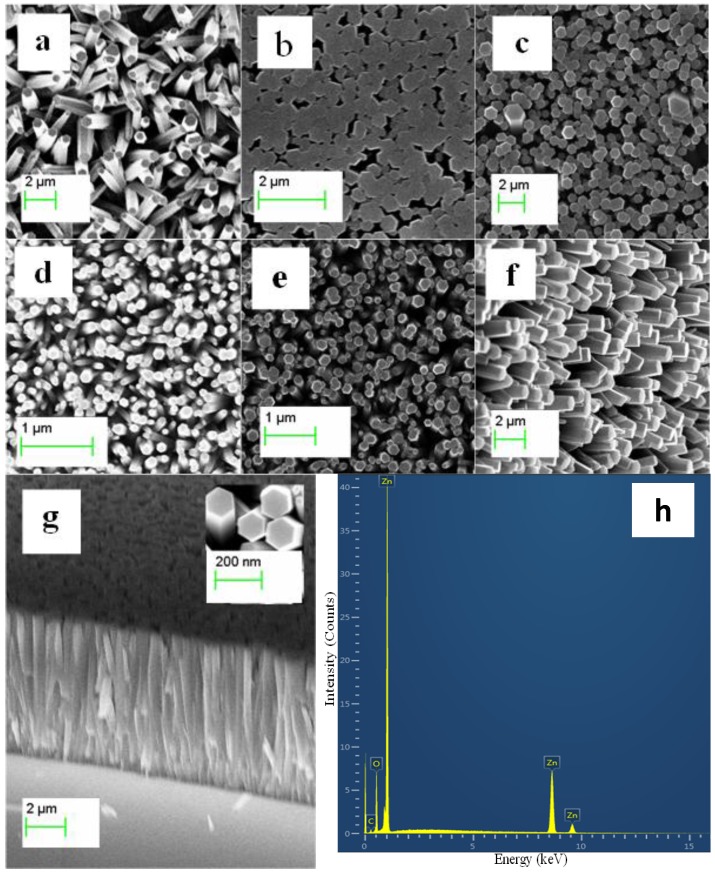
The FESEM image of the ZnO nanorods grown using a seed solution of chitosan with different amounts of ZnO nanoparticles. (**a**) 0; (**b**) 10; (**c**) 30; (**d**) 50; (**e**) 70; (**f**) 90; (**g**) the cross-sectional image of the ZnO nanorods grown using the seed solution (90 mg of ZnO nanoparticle); and (**h**) The EDX spectrum of the ZnO nanorods grown by using the seed solution of ZnO nanoparticles containing 70 mg of ZnO nanoparticles in the chitosan solution.

The field emission scanning electron microscopy (FESEM) study was carried out for the fabricated ZnO nanorods on a gold-coated glass substrate with different composite seed layers of ZnO nanoparticles/chitosan, as shown in Figure 2a–f. It has been reported that the seed layer contributes to the growth of highly oriented ZnO nanorods on the substrate [28,29,30,31,32]. This contribution can be inferred from Figure 2a, which shows the growth pattern of the ZnO nanorods on a seed layer of only chitosan. These results show the random growth of the ZnO nanorods with a low yield. However, when 10 mg of ZnO nanoparticles is used in the chitosan solution, an improved alignment in the ZnO nanorods is achieved, as shown in Figure 2b. Furthermore, when the amount of ZnO nanoparticles in the chitosan solution is increased to 30 mg, a better alignment of the ZnO nanorods with high density on the gold-coated glass substrate is observed, as shown in Figure 2c. A similar growth trend was observed for the composite seed layers prepared with 50, 70, and 90 mg of ZnO nanoparticles in the chitosan solution, as shown in Figure 2d–f. This growth pattern might be due initially to the uniform distribution of the ZnO nanoparticles in the chitosan solution and, subsequently, to the substrate-provided well nucleation sites for the synthesis of well-aligned and controlled ZnO nanorods. In addition to the top view FESEM images, a cross-sectional FESEM image was taken to confirm the growth pattern of the ZnO nanorods on the surface of the substrate, as shown in Figure 2g. It can be observed from this figure that the ZnO nanorods are 99% perpendicular to the substrate, and the measured length of the nanorod was approximately 7.1 µm with average diameter of 100 nm. The use of the composite seed layer of ZnO nanoparticles and chitosan has suggested a dual advantage of a seed layer composite: one advantage is the nucleation provided by the Zn and O ions of the ZnO nanoparticles, and the other advantage is that chitosan causes a uniform distribution of these nanoparticles on the substrate. Moreover, a combined cluster of the ZnO nanoparticles on the substrate [16] might be responsible for the number of ZnO nanorods with excellent alignment and density. Figure 2h shows the energy-dispersive x-ray (EDX) study of the ZnO nanorods fabricated using the composite seed layer of ZnO nanoparticles/chitosan. It can be observed from Figure 2 that the nanorods are only composed of Zn and O atoms, however, some amount of carbon atoms also appears in the graph, which may be due to the presence of carbon in chitosan. Chitosan is composed of carbon, hydrogen, oxygen, and nitrogen atoms, but these elements do not appear in the EDX graph because of the low percentage of these atoms in the chitosan molecule.

**Figure 3:**
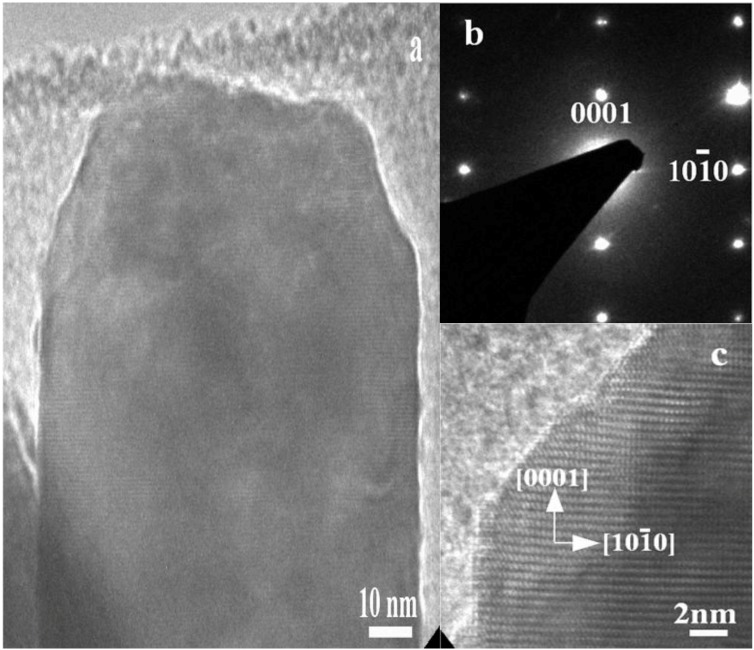
The HRTEM image of the ZnO nanorod grown with the seed solution of 70 mg of ZnO nanoparticles present in the chitosan solution.

The experimental results of the high-resolution transmission electron microscopy (HRTEM) analysis and selected-area electron diffraction for a single crystal ZnO nanorod are shown in Figure 3a. The HRTEM image indicated that the as-obtained ZnO nanorod is a single crystal with a wurtzite crystal structure and that the growth direction is along the (001) plane, as shown in Figure 3b. The HRTEM results obtained are in good agreement with the XRD results. The HRTEM study demonstrated that the ZnO nanorod exhibits a more pronounced lattice spacing of 26 Å, which is correlated with the (002) lattice spacing of the hexagonal structure of a crystalline ZnO nanorod, as shown in Figure 3c. The diameter of nanorod observed by the HRTEM analysis is about 90 nm, which is relatively comparable to the diameter measured from FESEM analysis. This analysis revealed the same results as the XRD analysis did, which indicates the single crystal nature of the fabricated ZnO nanorods and the preferred orientation of growth along the *c*-axis when using the composite seed layer of ZnO nanoparticles and chitosan.

### 2.3. The Atomic Force Microscopic Study of the Composite Seed of ZnO Nanoparticles and Chitosan

**Figure 4:**
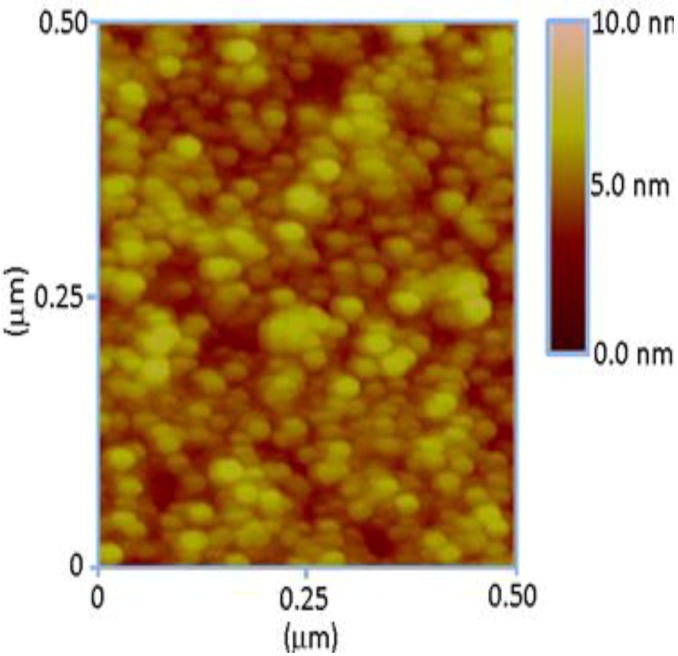
The AFM image of the 70 mg ZnO nanoparticles present in the chitosan solution.

A comfortable and easy heterogeneous nucleation on other surfaces has been reported in previous work [19], however, nucleation on a used substrate by providing a seed layer of ZnO nanoparticles is a simpler and more suitable method. This approach of using ZnO nanoparticles as a seed layer prior to the growth of ZnO nanorods has a direct effect on the morphology of the nanorods. An effective way to control the alignment and diameter of the fabricated ZnO nanorod arrays is always appreciated. Therefore, in the present study, an approach with a seed layer coating was used for the fabrication of well-aligned ZnO nanorods with a controlled diameter. The seed layer used in this study is a composite of freshly prepared ZnO nanoparticles and chitosan. The composite seed layer of ZnO nanoparticles and chitosan that was deposited on the substrate prior to the growth of the ZnO nanorods and was examined by atomic force microscopy (AFM), as shown in Figure 4. It can be inferred from Figure 2 that the ZnO nanoparticles are very well dispersed on the substrate and that the distribution of the nanoparticles on the surface is almost uniform with good nucleation sites for the controlled growth of ZnO nanorods.

### 2.4. The (Fourier Transform Infrared Spectroscopy) FTIR Study of the Fabricated ZnO Nanorods

**Figure 5:**
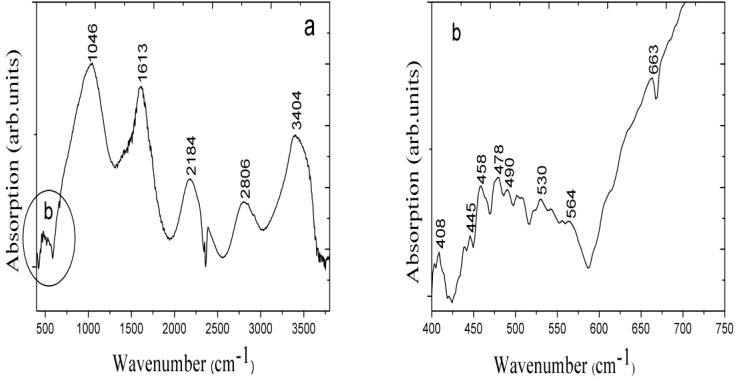
The FTIR spectrum of the ZnO nanorods grown with the seed solution of 70 mg of ZnO nanoparticles present in the chitosan solution at different frequency ranges (**a**) 400–4000 cm^−1^; and (**b**) 400–750 cm^−1^.

The FTIR experiment of the fabricated ZnO nanorods was performed in two different frequency ranges, as shown in Figure 5a,b. Figure 5a shows the spectrum measured in the 400–4000 cm^−1^ range at room temperature. The peak at 3404 cm^−1^ is attributed to vibrations of the O–H group and it is due to the absorption of water molecules during the growth time; the peak at 2806 cm^−1^ may be due to the C–H stretching mode. The peak at 1613 cm^−1^ is related to the C=O stretching vibration and that at 1046 cm^−1^ corresponds to the C–O stretching mode. Additionally, in Figure 5b, the FTIR spectrum in the 400–750 cm^−1^ range is shown, and characteristic peaks for the Zn–O modes are observed. Peaks at 406–512 cm^−1^ are characteristic of ZnO [33], and we observed peaks at approximately 408–530 cm^−1^ that can be assigned to the Zn–O stretching vibration modes of the ZnO nanorods.

### 2.5. Raman Spectroscopic Study of the As-Synthesized ZnO Nanoparticles

**Figure 6:**
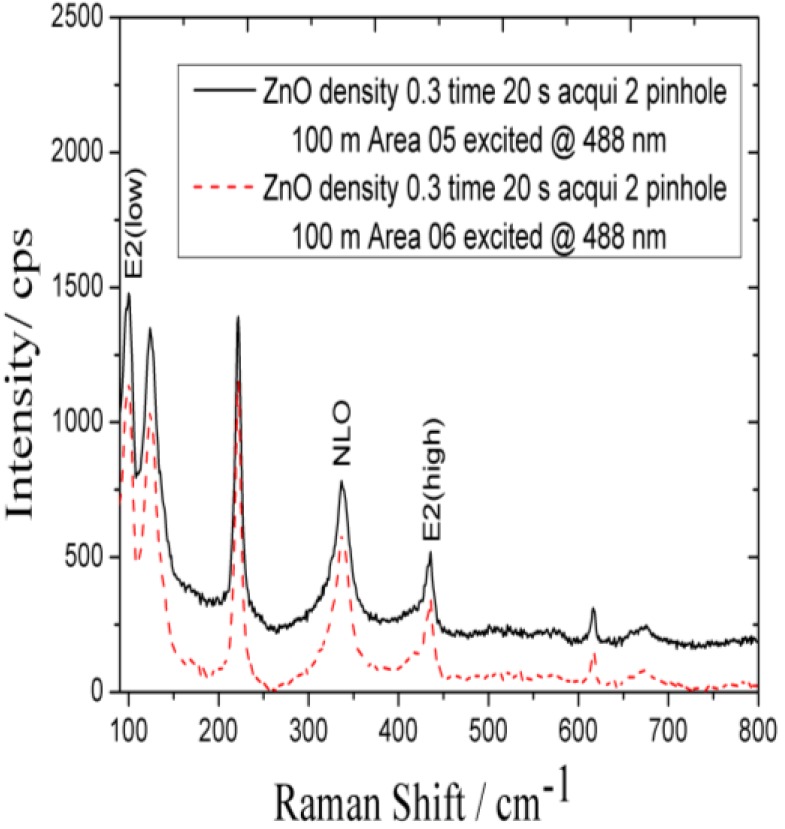
The Raman spectrum of the ZnO nanoparticles at room temperature at 488 nm.

The Raman study was carried out for the characterization of synthesized ZnO nanoparticles and Raman spectrum is shown in Figure 6. The characteristic ZnO nanoparticles peaks were observed in the Raman spectrum at 220, 323, 437, and 620 cm^−1^. The peak at approximately 332 cm^−1^ can be assigned to the second-order structure of ZnO, and the peak at 437 cm^−1^ is attributed to the E2 mode.

### 2.6. The Photoluminescence Study of the ZnO Nanoparticles/Chitosan Composite Seed Layer-Based ZnO Nanorods

**Figure 7:**
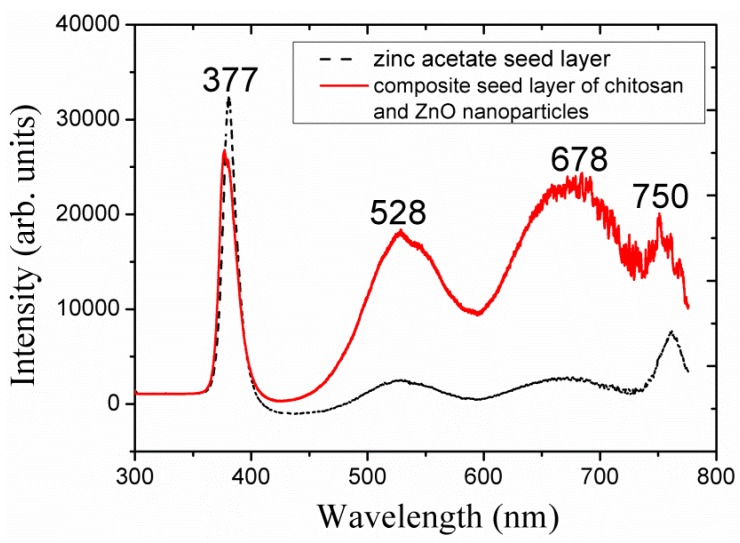
The photoluminescence (PL) spectrum of the ZnO nanorods grown with the seed solution of 70 mg of ZnO nanoparticles present in the chitosan solution and with the seed layer of zinc acetate dihydrate.

A photoluminescence study was carried out for the ZnO nanorods that were fabricated using the composite seed layer of ZnO nanoparticles/chitosan at room temperature, and the obtained results are shown in Figure 7.

Three types of peaks can be observed in Figure 7: a strong UV peak is observed at 377 nm; a green emission peak appeared at 528 nm; and orange/red emission peaks are observed at 676 and 750 nm. The broader green emission peak can be assigned to oxygen vacancies [34], and the broader orange/red emission peak may be due to the interstitial atomic defects in the ZnO [16]. A PL spectrum for the ZnO nanorods grown with the seed layer of zinc acetate dihydrate is shown with dotted lines for the comparison. From the PL spectra it is observed that the grown ZnO nanorods with a composite seed layer of ZnO nanoparticles/chitosan exhibited more defect levels compared to the ZnO nanorods grown with seed layer of zinc acetate dihydrate. Therefore, ZnO nanorods grown with a composite seed layer of ZnO nanoparticles/chitosan exhibit intense luminescence properties in the visible region.

## 3. Materials and Experimental Section

### 3.1. Chemicals Used

Zinc nitrate hexahydrate, hexamethylenetetramine, acetic acid, chitosan, lithium hydroxide monohydrate, zinc acetate dihydrate, and ethyl alcohol were purchased from Sigma Aldrich (Stockholm, Sweden) and were used without further purification.

### 3.2. Preparation of the ZnO Nanoparticles

The ZnO nanoparticles were prepared according to previously described methods [35]. Briefly, zinc acetate dihydrate was dissolved in 75 mL of ethyl alcohol and mixed with the ethanolic solution of lithium hydroxide monohydrate (LiOH·H_2_O) at room temperature with continuous stirring for approximately 8 hours. The role of lithium hydroxide monohydrate (LiOH·H_2_O, molecular weight = 41.96 was used to hydrolyze the precursor. The mixing of LiOH to the transparent precursor leads to the formation of ZnO nanoparticles sol along with the reaction products like lithium acetate and H_2_O through hydrolysis. Presence of water plays an important role in growth of ZnO nanoparticles, and therefore presence of water is strictly controlled during the reaction and during precipitation to obtain nanopowder. The prepared ZnO nanoparticles were retrieved from the colloidal solution using hexane as a precipitating agent. Finally, the obtained product was dried under vacuum conditions.

### 3.3. Preparation of the Composite Seed Solution of ZnO Nanoparticles and Chitosan

Different composite seed solutions of ZnO nanoparticles and chitosan were prepared by mixing 10, 30, 50, 70, and 90 mg of the ZnO nanoparticles in the chitosan solution. The chitosan solution was prepared by dissolving 35 mg of chitosan in 1% acetic acid. A homogeneous seed solution was obtained by sonication. Additionally, a chitosan solution without ZnO nanoparticles was used as a seed layer to confirm the role of the ZnO nanoparticles in the growth of the ZnO nanorods.

### 3.4. The Growth of the ZnO Nanorods on a Gold-Coated Glass Substrate

The ZnO nanorods were fabricated on a gold-coated substrate using the hydrothermal growth method, and each step of the growth is as follows:

A Satis evaporator (725) was used to coat the gold layer onto the glass substrates. The glass substrates were affixed in the Satis evaporation chamber, and a 20 nm thick adhesive layer of titanium for gold was deposited. Following this, a 100 nm thick gold layer was evaporated. Subsequently, the gold-coated glass substrates were washed with isopropanol for 10 minutes in an ultrasonic bath and cleaned with the deionized water. The substrates were then dried in air at room temperature. The substrates were spin coated with the composite seed layer of ZnO nanoparticles and chitosan 2 to 4 times at 3000 rpm. The seed layer-coated substrates were annealed at 120 °C for 20 min, affixed in a Teflon sample holder and vertically dipped into an equimolar solution of 0.075 M zinc nitrate hexahydrate and hexamethylenetetramine. The growth solution containing the annealed substrates was kept in a preheated oven at 95 °C for 4 to 7 hours. Finally, after completion of the growth time, the substrates were removed from the oven and cleaned with deionized water to remove the solid residue particles from the surface of the ZnO nanostructures.

### 3.5. Characterization of the As-Synthesized ZnO Nanostructures

The synthesized ZnO nanoparticles were studied by Raman spectroscopy, and the composite seed layer-coated gold-coated glass substrate was studied by AFM. The AFM analysis was using Veeco Dimension 3100 (Veeco Instruments, Inc., Plainview, NY, USA), operation in tapping mode and the silicon tip (resistivity 0.01–0.025 Ω·cm; cantilever T = 3.95–4.71 μm; W = 29–31 μm; L = 124 μm; C = 39–71 N/m; and f_0_ = 330–399 kHz). The ZnO nanorods were characterized by FESEM that was performed using LEO 1550 Gemini, field emission gun was operated at 20 kV. The XRD scans (0.1/s) were carried out on Phillips PW 1729 powder diffractometer using the Cu Kα radiation (λ = 1.5418 Å) for the study of crystal arrays of ZnO nanorods. A HRTEM analysis was performed using an FEI Tecnai G2 TF20 UT (Hillsboro, OR, USA) with a field emission gun operating at 200 kV and a point resolution of 1.9 Å and equipped with an EDX. A FTIR was used for the investigation of the Zn–O bonding. An optical study was performed using a PL technique at room temperature. In the photoluminescence experiment third harmonics (λ_e_ = 266 nm) from a Coherent Ti: sapphire laser was employed and the detection was observed with Hamamatsu CCD camera. For the dispersion of PL signal a single monochromator of 1 m focal length (model Brucker Optics Chromex 25, Bruker Corp., Billerica, MA, USA) was associated to diffraction grating of 150 lines/mm.

## 4. Conclusions

In this study, ZnO nanorods were fabricated by a hydrothermal growth method using a composite seed layer of inorganic and organic materials. The seed layer was composed of the inorganic ZnO nanoparticles and the organic chitosan-conducting polymer. Different composite seed layers were prepared and used for the synthesis of the ZnO nanorods. FESEM, EDX, HRTEM, XRD, and FTIR techniques were used for the structural characterization of the ZnO nanorods, and these experiments explored the improved alignment, high density, and c-axis orientation of growth of the ZnO nanorods. Moreover, a PL study was used to determine the optical properties of these materials, and the measured results are consistent with the XRD results. This study has provided an excellent way to fabricate ZnO nanorods with excellent alignment and proper orientation relative to the substrate using a low-temperature, low-cost, simple and aqueous chemical growth method those results in a high yield of the desired nanomaterial. The obtained results indicate that the use of this method can potentially increase the performance of optoelectronic devices based on ZnO nanorods on the nanoscale, where alignment of nanostructures has significant contribution.
